# Valosin-containing protein (VCP), a component of tumor-derived extracellular vesicles, impairs the barrier integrity of brain microvascular endothelial cells

**DOI:** 10.1016/j.bbadva.2024.100130

**Published:** 2024-12-12

**Authors:** Ramon Handerson Gomes Teles, Nicolas Jones Villarinho, Ana Sayuri Yamagata, Camila Tamy Hiroki, Murilo Camargo de Oliveira, Gisela Ramos Terçarioli, Ruy Gastaldoni Jaeger, Patrick Meybohm, Malgorzata Burek, Vanessa Morais Freitas

**Affiliations:** a University of São Paulo, Department of Cell and Developmental Biology, Institute of Biomedical Sciences (ICB), São Paulo, 05508-000, Brazil; b University Hospital Würzburg, Department of Anaesthesiology, Intensive Care, Emergency and Pain Medicine, 97080 Würzburg, Germany; c University Würzburg, Graduate School of Life Sciences, Campus Hubland Nord, 97074 Würzburg, Germany

**Keywords:** Extracellular vesicles, Exosomes, Microvesicles, Breast cancer, Metastasis, hCMEC/D3, VEGFR2, Valosin-containing protein, VCP

## Abstract

Metastases are the leading cause of cancer-related deaths, and their origin is not fully elucidated. Recently, studies have shown that extracellular vesicles (EVs), particularly small extracellular vesicles (sEV), can disrupt the homeostasis of organs, promoting the development of a secondary tumor. However, the role of sEV in brain endothelium and their association with metastasis related to breast cancer is unknown. Thus, this study aimed to investigate sEV-triggered changes in the phosphorylation state of proteins on the surface of brain endothelial cells, as they form the first barrier in contact with circulating tumor cells and EVs, and once identified, to modulate its interactors and effects from this through different functional assays. We used the most aggressive breast cancer cell line, MDA-MB-231, and its brain-seeking variant, MDA-MB-231-br. From these cells, small and large extracellular vesicles were harvested to treat hCMEC/D3 cells, an immortalized cell line from the human brain microvasculature. Higher levels of phosphorylation of VEGFR1 and VEGFR2 were found in hCMEC/D3 cells treated with MDA-MB-231-br sEV. By computational analysis, the Valosin-Containing Protein (VCP) was predicted to be an important sEV cargo affecting the VEGFR2 intracellular trafficking, validated by western blotting analysis. Then, VCP was modulated by cell transfection or chemical inhibition in hCMEC/D3 cells and assessed in different functional in vitro assays evidencing a significant effect on the functionality of these cells. Thus, this study demonstrates that the VCP-containing sEVs induce modifications at different phosphor sites of VEGFR2 and effectively modulate the state of brain microvascular endothelial cells.

## Introduction

Breast cancer is the most prevalent type of oncological disorder in women, accounting for two million new cases worldwide each year [1]. The main cause of cancer-related death is metastasis formation, accounting for 90 % of them [2]. Metastatic cells follow a well-known route called metastatic cascade as they spread throughout the body [3]. However, before its invasion, a circulating tumor cell (CTC) must also interact with endothelial cells, which are highly specific for each organ, making them morphologically and biologically heterogenous [4,5]. In addition, organs can contain a specialized vasculature, such as the blood-brain barrier (BBB) [6].

The brain is one of the secondary sites of metastasis of breast cancer, once established it becomes a challenging treatment resulting in a high lethality index, accounting for more than 30 % of cases in autopsy studies [[6], [7], [8]]. Furthermore, among the subtypes of breast cancer, triple-negative breast cancer (TNBC), which lacks functional hormonal receptors, has the shortest median survival time of only 5 months [9]. Therefore, TNBC is related to most observed brain metastases, presenting aggressive cells capable of inducing disruption of the BBB, allowing them to migrate through it [3,10].

However, BBB disruption may occur not only when a CTC is in direct contact with the endothelial cells, but also before the CTC leaves the primary tumor, i.e., without intravasation. This assumption is based on recent findings showing that an organ can be prepared to receive a CTC when exposed to tumor-derived small extracellular vesicles (sEV), facilitating its extravasation [11,12]. Thus, cellular communication between distant cells can occur through signaling factors carried by structures released from malignant to healthy cells, such as the extracellular vesicles (EVs), called sEV and large EVs [13,14].

Small EVs have a multivesicular endosomal origin ranging from 40-150 nm, while large EVs differ by size, ranging from 100-1000nm and originating from the budding and blebbing of the plasma membrane [15]. However, both types of EVs are known to play significant roles in cellular communication by transferring functional biological molecules, such as miRNAs, mRNA, and proteins [[16], [17], [18]]. The EVs are continuously released by all cells, reflecting the nature of the donor cell and its physiological state while affecting the receptor cell [[19], [20], [21], [22]]. Therefore, it is important to understand how the EV cargoes of a tumor affect the recipient cells and favor the metastasis formation.

Regarding brain metastasis, its formation occurs in a much more complex scenario due to the complex BBB structure consisting of brain microvascular endothelial cells, pericytes, and astrocytes. The endothelium forms tight junction complexes that regulate the paracellular permeability [23]. Studies have suggested that tumor-derived sEV can contribute to BBB leakage and subsequent invasion of the CTC to the brain [24,25]. However, which signaling pathways are modulated in the brain endothelium remains to be elucidated.

Thus, this study focused on identifying proteins that are modulated by the interaction between EVs from TNBC cells and the immortalized brain microvascular endothelial cell line hCMEC/D3. This cell line is one of the widely used in vitro BBB models with proven expression of BBB-specific markers and well-established BBB properties [[26], [27], [28]]. We demonstrate that hCMEC/D3 cells treated with tumor EVs carrying Valosin-Containing Protein (VCP) have a distinct VEGFR2 phosphorylation, while VCP modulation in hCMEC/D3 cells by transfection resulted in changes in barrier properties. VCP is a hexameric AAA+-type ATPase assisted by nearly 30 cofactor proteins that act in different pathways [29], and VEGFR2 is a receptor tyrosine kinase that has important roles in angiogenesis, vascular basement membrane degradation, migration, and proliferation of endothelial cells [30].

## Materials and methods

### Cell culture, EV isolation and characterization, and functional assays

#### Cell lines

MDA-MB-231 parental cells (MDA-par) were obtained from the ATCC collection and its brain-seeking variant expressing green fluorescence protein, MDA-MB-231-br-GFP (MDA-br), was kindly donated by Prof. Patricia Steeg (Center for Cancer Research, NCI) [31], both cell lines were grown in Dulbecco's modified Eagle's medium-High Glucose (DMEM-HG, Sigma-Aldrich, #56499C), with 5 % fetal bovine serum (FBS, Thermo Fisher Scientific, #26140079), 100U/mL penicillin, and 100 µg/mL streptomycin (Merck, Sigma-Aldrich, #P4333). hCMEC/D3 cells from two different sources were used, one from BCRJ (Banco de Células do Rio de Janeiro, Brazil) and grown in the basal medium EGM (Lonza, CC-3156), supplemented with the microvasculature bullet kit (Lonza, #CC-4147), and other from Germany grown in Endothelial Cell Basal Medium (Pelo Biotech, PB-BH-100-9806)[28]. hCMEC/D3 cells were cultured in plates pre-coated with Collagen Type I for 1h at 4 °C. Afterward, the solution was removed, and the plates were kept in the incubator for temperature adjustment. All cell lines were cultured under standard conditions in a humidified incubator containing 5 % CO_2_ at 37 °C.

### Isolation and characterization of extracellular vesicles

Breast cancer cell lines were cultured until reaching 80 % confluency, then, the monolayer was gently rinsed twice with sterile phosphate-buffered saline (PBS) to remove excess FBS, and a fresh DMEM medium depleted of FBS was added. After 24h, the conditioned medium was harvested by pelleting cell debris at 2000 × g for 10 min at 4 °C. Following a new centrifugation at 15000 × g for 30 min at 4 °C, large EVs were collected and recentrifuged in PBS filtered (0.2µm) to remove the medium. Finally, the conditioned medium was ultracentrifuged at 100.000 × g for 2 h10 min at 4 °C, and the pelleted sEV was resuspended in PBS and ultracentrifuged again to remove residual media. Ultracentrifuge steps were carried out in a Beckman L8-80M (Beckman CoulterTM), k-factor of 63, using the rotor Type 60 Ti. Large EV and sEV were resuspended in 50µL of PBS and stored at -20 °C [32].

### Biochemical and physical characterization of extracellular vesicles

Initially, using Western blot, 1 × 10^6^ cells of MDA-par and MDA-br, their sEV and large EV were lysed in an appropriate volume of 5 × RIPA buffer (50nM Tris-HCl pH 7.4, 750 mM NaCl, 0.5 % Sodium Deoxycholate, 0.5 % SDS, 5 % Triton, 1mM Sodium Orthovanadate, 50 mM NaF, 5µg/ml Pepstatin, 5 µg/ml Aprotinin, 5 µg/ml Leupeptin) on ice for 15 min followed by centrifugation at 10,000 × g for 10 min at 4 °C. The supernatant was collected and protein concentrations were determined by BCA assay (ThermoFisher Scientific, #23225), according to kit instructions.

An equal amount of 5µg of protein from each EV lysate was mixed with 4 × Laemmli buffer (750mM Tris-HCl pH6.8, 5 % SDS, 40 % glycerol, and 80 mM DTT) and heated to 95 °C for 5min. The protein was loaded on 10 % polyacrylamide-resolving gels and ran at 70 V for 120 min. Resolved proteins were transferred to 0.22 µm nitrocellulose membrane, for 16h at 4 °C. The membranes were blocked for 1h at RT in 1 × TBS containing 5 % Bovine Serum Albumin (BSA). Proteins were detected by incubation with the following primary antibodies (1:1000): Calnexin (CST, #2679), CD63 (CST, #55051), CD9 (CST, #98327), TSG101 (CST, #72313), β-actin (Sigma-Aldrich, #A5316), diluted in blocking solution (TBST 1 % in BSA 5 %) for 16h at 4 °C. Following four 10min washes of TBS with 0.5 % Tween-20 (TBS-T), membranes were incubated in appropriate dilution (1:1000) with anti-rabbit (CST, #7074) or anti-mouse (CST, #7076), diluted in blocking solution for 1h at RT. The membranes were rewashed four times for 10 min each, incubated in ECL (Biorad, Cat. 170561) and the bands were visualized on an Amershan^TM^ Imager 600.

The procedures concerning Nanoparticle Tracking Analysis (NTA) were made using a NanoSight NS300 system (Malvern Technologies, Malvern, UK), in which the particle number and size distribution were determined using samples diluted (1:1000) in PBS filtered twice (0.2µm).

### Effect of EVs on hCMEC/D3 cell viability

Viability assays were performed as first described by Mosmann [33]. hCMEC/D3 were seeded (1 × 10^4^ cells/well) into 96-well plates, waiting to reach confluency, and treated with different concentrations of exosomes and microvesicles (0.5, 1, 2, 4, 8µg/ml) for 24h. After that, the medium was removed, and a solution of MTT (1µg/ml) was added for 4h. This solution was removed and 100µL of DMSO/well was used to solubilize formazan crystals. The absorbance was measured on an ELISA plate reader (EPOCH, Bio Tek) at a wavelength of 570nm.

### EV-induced phosphorylation of receptor tyrosine kinases in hCMEC/D3

A set composed of 49 human phosphorylated proteins was evaluated using the Proteome Profiler^TM^ Array Human Phospho-RTK Array Kit (R&D Systems, Minneapolis, MN, USA, Cat. ARY001B) according to the manufacturer's instructions. First, hCMEC/D3 cells were treated with 1 µg/ml of sEV from MDA-par (sEV-par) or MDA-br (sEV-br) for 24h. Thereafter, the cells were harvested in the lysis buffer provided with the kit, the protein concentration was measured, and 300 µg was loaded onto membranes coated with phosphokinase antibodies. Detection was performed with Amersham^TM^ Imager 600 (GE Healthcare).

### Bioinformatic analysis of protein interactions and clinical relevance

Proteomic datasets on breast cancer or extracellular vesicles (Table 1) were downloaded from the PRIDE database [34], while the dataset on brain microvasculature was downloaded from a supplementary material of Ito, et al. [35].

**Table 1:** Public proteomics datasets analyzed, available in the PRIDE database or as a source article-supplementary material.

**Project Number (PRIDE)**	**Project title**	**Sample used**	**Ref**
PXD012162	Proteomic profiling of breast cancer-derived extracellular vesicles	Cell lysate of extracellular vesicles from MDA-MB-231	[55]
PXD005719	Comparative membrane proteomics analyses of breast cancer cell lines to understand the molecular mechanism of breast cancer brain metastasis	Cell membrane proteins of MDA-MB-231-wt and MDA-MB-231-br	[56]
Supplementary Material	Identification of cell-surface proteins endocytosed by human Brain Microvascular Endothelial Cells in vitro	Cell membrane endocytosed into brain microvascular endothelium	[35]

First, the raw MS files were searched with the MaxQuant Software (Version 2.0.3) [36]. MS/MS spectra were searched by Andromeda against a reviewed *homo sapiens* database (https://www.uniprot.org/proteomes/UP000005640, 20,168 entries). Based on the statistical guide workflow to Microsoft Excel [37], raw intensities extracted by MaxQuant were log2 transformed, and normalized by median. Imputation of missing values was carried out by Probabilistic Minimum Imputation, and p-value calculated applying Shapiro-Wilk test followed by Mann-Whitney. Finally, the p-values were transformed into -log2, and the fold-change values were corrected using Bonferroni test.

Next, protein names were identified using UniProt ID converter [38] and the shared protein among breast cancer cell lines, extracellular vesicles, and brain endothelium were analyzed in an online Venn diagram maker, Venny 2.1 [39]. These proteins were crosslinked with the pathways belonging to the receptor tyrosine kinase identified by the Human Phospho-Tyrosine Kinase Array, using the Reactome database [40]. Concomitantly, the protein fold-change of each dataset was displayed in a Volcano Plot created at VolcaNoseR [41].

The Overall Survival plot was created using clinical datasets [[42], [43], [44], [45], [46]] deposited in the cBioPortal [47,48], covering a total of 3065 patients over a 30-year follow-up.

RNA-seq datasets available at The Human Protein Atlas [49] were analyzed following the Integrated RNA-Seq Analysis Pipeline (iRAP 1.0.1). Coding RNA from tissue samples of 122 human individuals were first filtered to discard reads of low quality, bacterial contamination, or uncalled characters, after being compared to a genome reference (Ensembl release: 95, HISAT2 version: 2.1.0) followed by gene and transcript quantification using FeatureCounts (v. 1.6.2) and Kallisto (v. 0.42.4). Normalized Counts per Gene (TPMs) were calculated from the raw counts. These were averaged for each set of technical replicates, and then quantile normalized within each set of biological replicates using limma. All the pipeline, analysis, and raw datasets are registered in ArrayExpress database [50] with the accession code E-MTAB-2836.

### Western blot analysis of VEGFR2 and associated proteins

The hCMEC/D3 cells were cultured in 100 mm-plate dishes until reached monolayer confluence. Then, they were treated or not with EV, harvested, and lysed as described above.

An equal amount of 10 µg of protein from each lysate was mixed with 4 × Laemmli buffer and heated to 95 °C for 5min. Thus, the protein was loaded on 10 % polyacrylamide resolving gels and run at 70V for 120min. Resolved proteins were transferred to 0.22µm nitrocellulose membrane, for 16h at 4 °C. The membranes were blocked for 1h at RT in 1 × TBS containing 5 % BSA.

Alternatively, hCMEC/D3 cells were lysed using RIPA as already described, and 10 μg of total protein were loaded on Novex^TM^ Tris-Glycine Mini Protein Gels (4-12 %), and run at 150V for 1h, as recommended by the company. Resolved proteins were transferred to 0.22μm PVDF membrane, for 16h at 4 °C. The membranes were blocked for 1h at RT in 1 × TBS containing 5 % BSA.

Proteins were detected by incubation with the following primary antibodies: VEGFR2, p-VEGFR2-Tyr951, Tyr996, Tyr1059, Tyr1175 from a sampler kit (Cell Signaling Technology (CST), #12599), Rab4A, Rab5A, Rab7, Rab11 from a sampler kit (CST, #9385), VEGFR1 (CST, #2893), VCP (CST, #2648), EEA1 (CST, #3288), LAMP1 (CST, #9091), Actin-Beta POD (Sigma, #A3854), Caspase 3 (CST, #96625), Phalloidin (Actin-satin 488) (Cytoskeleton Inc, #PHDG1), in blocking solution (TBST 1 % in BSA 5 %) for 16h at 4 °C. Following four 10min washes of TBS with 0.5 % Tween-20 (TBS-T), membranes were incubated in appropriate dilution (1:1000) with Anti-rabbit (CST, #7074) or Anti-mouse (CST, #7076), diluted in 5 % blocking solution for 1h at RT. The membranes were washed again four times for 10 min each, incubated in ECL (Biorad, Cat. 170561) and the bands were visualized on an Armshan^TM^ Imager 600.

### Modulation of VCP expression in hCMEC/D3 cells via transfection

The hCMEC/D3 cells were seeded at 1 × 10^5^ cells in a 6-well plate previously coated with collagen type I and allowed to grow to 70 % confluency at the time of transfection. The plasmid VCP CRISPR Activation Plasmid (h) (sc-401052-ACT, Santa Cruz Biotechnology) was mixed with Lipofectamine^TM^ 3000 (L3000001, ThermoFisher Scientific) and prepared according to the manufacturer's instructions. The solutions were combined, vortexed, and incubated for the recommended duration to facilitate complex formation. Subsequently, the transfection mixture was added to the cells and incubated for 24 h. Following incubation, the cells were washed with PBS and a fresh medium was added. The experiments to analyze influence of VCP activation on endothelial cells were conducted from the third to the fifth day post-transfection.

### Gene expression analysis of BBB-related genes

For Real-Time RT-qPCR, total RNA was isolated from hCMEC/D3 cells using the kit NucleoSpin RNA (#740955.250). 1μg of RNA isolated was used to synthesize cDNA by the kit MultiScribe^TM^ Reverse Transcriptase (ThermoFisher Scientific, #4311235). The amplification was performed after an initial warm-up phase of 10 min at 25 °C for optimal Master Mix activity and 120 min at 37 °C completing forty amplification cycles. The cDNA was subjected to reverse transcription-PCR quantification using commercially available TaqMan probes (FAM labeled; Applied Biosystems) on a QuantStudio^TM^ 7 Flex Real-Time PCR System (Cat.: 4485701). For this, 7.5 μl of PCR Master Mix, 2.5 μl of cDNA, and TaqMan probes with a FAM reporter dye at the 5’ end and a nonfluorescent quencher at the 3’ end were used. Samples were normalized to β-actin Ct values for each experiment. The following TaqMan probes were used (Table 2):

**Table 2:** TaqMan probes used for Real-Time RT-qPCR assay.

Assay ID	Gene
Hs00997642_m1	VCP
Hs00911700_m1	KDR
Hs00243202_m1	S100A4
Hs01102463_m1	AHNAK
Hs00968305_m1	MMP-3
Hs00957562_m1	MMP-9
Hs00165949_m1	TIMP3
Hs00533949_s1	Claudin5

### Effect of VCP modulation on hCMEC/D3 cell proliferation

The hCMEC/D3 cells, both transfected and parental, were seeded at a density of 1 × 10^4^ cells/well in a 96-well plate and allowed to grow for 24 h. Following this incubation period, the protocol was executed according to the manufacturer's recommendations (BrdU cell proliferation assay, #QIA58-200test). In summary, the medium was aspirated, and 20 μL of 5-bromo-2′-deoxyuridine (BrdU) solution was added to each well and incubated with the cells for 16 h. Subsequently, the cells were fixed, and anti-BrdU antibody was applied and allowed to incubate for 1 h. After incubation with the antibody, the cells were washed to remove excess reagents, and a solution was added to facilitate the detection of BrdU incorporation, followed by measurement of absorbance at 492 nm using a microplate reader. The extent of cell proliferation was then assessed by comparing the absorbance readings between the transfected and parental cell groups.

### Impact of VCP modulation on angiogenesis in hCMEC/D3 cells

Matrigel basement membrane matrix (Corning, Bedford, MA, USA) was thawed from -20 °C by gradual warming overnight at 4 °C and subsequently kept on ice until needed. Using pre-cooled pipet tips, 10μl of Matrigel was dispensed into each well of a μ-Slide 15 well 3D bitrate (#81506) chamber. This was incubated at 37 °C for 30 min to allow the Matrigel to solidify. Afterward, the hCMEC/D3 cells (1 × 10^4^ cells in 50 μl of medium) were added to each well containing the solidified Matrigel. The chamber was returned for the incubator, and at different time points images of the tube formation assay were captured using a phase contrast microscope EVOS^TM^ XL Core (Invitrogen, #AMEX1100). The ImageJ plugin “Angiogenesis Analyzer for ImageJ” was employed for quantitative analysis of the tube length and other parameters.

### Effect of VCP modulation on hCMEC/D3 cell migration

Culture inserts (Ibidi, Cat.: 81176) were used to assess cell migration, in which each well received 8 × 10^4^ cells in 80μl of medium. Following 24 h for cell attachment, the cells were treated with mitomycin-C (5μg/ml) (Merck, #M4287), for 2 h, to halt cell proliferation. Subsequently, a cell-free gap of 500 μm was created by removing the culture insert. Images were captured in different time points, being 2, 4, 6, and 24h, using an inverted phase-contrast microscope EVOS^TM^ XL Core (Invitrogen, #AMEX1100), and the percentage of wound closure was determined using an ImageJ plugin [51].

### Assessment of BBB permeability upon VCP modulation

The hCMEC/D3 cells were cultured at a concentration of 1 × 10^5^ cells/mL in the upper compartments of commercially available 12-well transwells (Costar, #3401) with 0.4μm diameter pores, which had been pre-coated with collagen Type I for 1 h at 4 °C. Upon reaching confluency, the media in the upper compartments were replaced with 500μL of HEPES-Ringer buffer mixed with Fluorescein Isothiocyanate (FITC) at a concentration of 1μM. Subsequently, the bottom compartment was filled with 1500 μL of HEPES-Ringer buffer, and the transwells were placed in an incubator for a total of 1 h. At 20 min intervals, a specified volume (200 μL) from the bottom compartment was transferred to a 96-well black plate clear bottom, and the corresponding fluorescence intensity was measured using an Infinite 200 Pro (TECAN). Molecular concentrations in the collected samples were then estimated based on the fluorescent intensity measurements using a calibration curve. A blank insert without a monolayer but coated with collagen type I was used for comparison. Both the initial concentration and incubation time were carefully selected to ensure that the concentration measurements remained within the linear range of the calibration curves, thereby avoiding signal saturation.

### Measurement of transendothelial electrical resistance (TEER)

The TEER measurement was conducted in the same transwell inserts used in the permeability assay, in which the preparation was already described. An ENDOHM12 EVM-EL-03-01-02 electrode attached to an EVOM^TM^-EVM-MT-03-01 (World Precision Instruments) manual Ohm meter was used for TEER measurement. Values were normalized considering resistance/cm², based on “Resistance (Ω) × Effective Membrane Area (πr²)”.

### Breast cancer cell transmigration across the hCMEC/D3 monolayer

Initially, the transwell inserts with 8μm pores (Corning, #3422), kept in a 12-well plate, were coated with collagen type I for 1 h. Following this, hCMEC/D3 cells were seeded onto the upper insert at a density of 1 × 10^5^ cells in 500 μl and allowed to grow until a confluent monolayer was formed (48 h later). Subsequently, breast cancer cells were stained with Cell Tracker Green (ThermoFisher Scientific, #CMFDA C2925) for 15 min, followed by washing of the plate and cell counting to determine the appropriate number for seeding into the top insert along with serum-free medium, where remained for 24h. Simultaneously, a medium containing serum was added to the lower chamber to create a chemotactic gradient.

In parallel, a known amount of stained breast cancer cells was utilized to generate a standard curve, facilitating subsequent quantification of transmigrated cells. After a 24 h incubation period, the tumor cells that had transmigrated to the bottom chamber were carefully collected and centrifuged at 4 °C and 300 × g. These cells were then transferred to a 96-well black plate with a clear bottom. The fluorescence was measured using a plate reader and the amount of transmigrated tumor cells was estimated using the standard curve.

### Statistical analysis

The experiments were repeated three times in triplicates. Data are presented as mean ± SEM or its transformation in Log 2 for better visualization and comprehension. Values were compared using one-way ANOVA followed by Tukey's multiple comparison *post-hoc* tests or t-test with Holm-Sidak correction, using GraphPad Prism 8.0 software (GraphPad Software Inc., San Diego, CA, USA). Changes were considered statistically significant at p ≤ 0.05.

## Results

### Characterization of extracellular vesicles from MDA-MB-231 cells

**Fig. 1:**
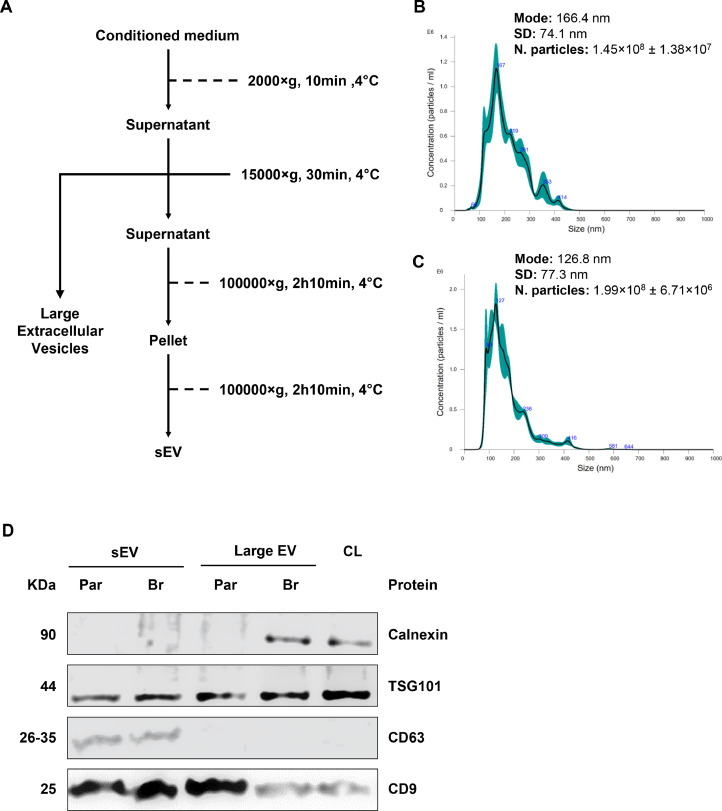
Schematic representation of EV isolation protocol and characterization of isolated EVs from MDA-MB-231 parental (MDA-par) and brain-seeking (MDA-br) cells. **(A)** Differential centrifugation scheme for obtaining EVs. **(B)** Nano-Tracking Analysis of small EV from MDA-MB-231-parental and **(C)** MDA-MB-231-brain variant, evidencing the size distribution as a function of particle concentration. **(D)** Western blot analysis of calnexin, TSG101, CD63, and CD9 in sEV, Large EV and Cell Lysate (CL). Data are presented as the mean ± SEM of three independent experiments (*n* = 3).

Initially, differential centrifugation was used to acquire small and large EVs from the parental TNBC and its brain-seeking variant, following the protocol schematized in Fig. 1A. The average size analyzed by NTA for MDA-par-sEV was 166.4 ± 74.1 nm (Fig. 1B), and 126.8 ± 77.3 nm for MDA-br-sEV (Fig. 1C), while the biomarkers identified by Western blot (Fig. 1D), calnexin, TSG101, CD63, and CD9, confirm each vesicle according with its subtype, also compared to the cell lysate (CL). The size range and the pattern of the expressed biomarkers are consistent with the Minimal Information for Studies of Extracellular Vesicles [52] and correlate with results from other studies [53,54].

**Fig. 2:**
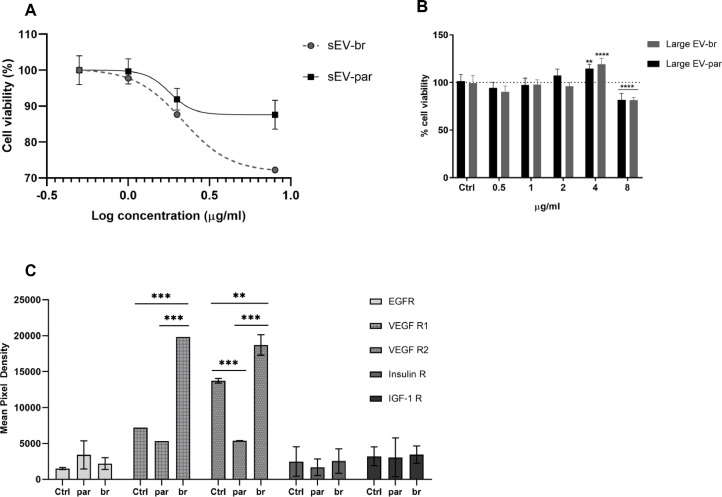
**(A)** Cell viability of hCMEC/D3 cells after 24h incubation with different concentrations of sEV isolated from MDA-MB-231-br (sEV-br) and MDA-MB-231-par (sEV-par) cells. **(B)** Cell viability of hCMEC/D3 cells after 24h incubation with different concentrations of large EVs isolated from MDA-MB-231-br (Large EV-br) and MDA-MB-231-par (Large EV-par) cells. Two-way ANOVA (Dunnett's multiple comparisons test), mEV-par (4µg/ml × Ctrl = adj. *p=* 0.0084; 8µg/ml × Ctrl = adj. *p* < 0.0001), mEV-br (4µg/ml × Ctrl, adj. *p* < 0.0001; 8µg/ml × Ctrl, adj. *p* < 0.0001). **(C)** Mean pixel density of phosphorylated receptor tyrosine kinases in hCMEC/D3 cells after 24h incubation with 1µg/ml of sEV-br or sEV-par or left untreated. Two-way ANOVA (Tukey's multiple comparisons test), VEGFR1 (Ctrl × sEV-br and sEV-par × sEV-br, adj. *p* < 0.0001), VEGFR2 (Ctrl × sEV-par, adj. *p* < 0.0001; Ctrl × sEV-br, adj. *p=* 0.0063; sEV-par × sEV-br, adj. *p* < 0.0001). Data from three independent experiments performed in triplicate (*n* = 9) in A and B, or three independent experiments (*n* = 3) in C.

Next, we performed the cell viability assay using the MTT method [33]. hCMEC/D3 cells were treated with different concentrations of extracellular vesicles for 24h, resulting in an IC_50_ of sEV-br = 1.798 µg/ml and sEV-par = 2.161 µg/ml, represented in the log scale on the graph as sEV-br = 0.2547, and sEV-par = 0.3347 (Fig. 2A). Large EV showed a different pattern, in which both Large EV-par and -br had increased and maximal activity at a concentration of 4 µg/ml (Fig. 2B).

Based on these results, we choose a sublethal concentration of small EV of 1 µg/ml to treat hCMEC/D3 to assess the modulation of 49 different phospho-Tyrosine Kinase Receptors, using the kit *Proteome Profiler^TM^ Array* (R&D System). Following the kit instructions, the endothelial cells were homogenized and a membrane was incubated overnight (16h) with the cell lysate. We identified VEGFR2 differentially phosphorylated, with sEV-par reducing the phosphorylation and sEV-br increasing it, compared to control. Similar results were obtained for VEGFR1 (Fig. 2C).

### Identification of key proteins and clinical significance of VCP

To assess the proteins that interact between breast cancer, hCMEC/D3 cells, and the small EVs (named exosomes in the original dataset), we first analyzed public proteomic datasets deposited in the PRIDE database [34], listed in Table 1.

**Fig. 3:**
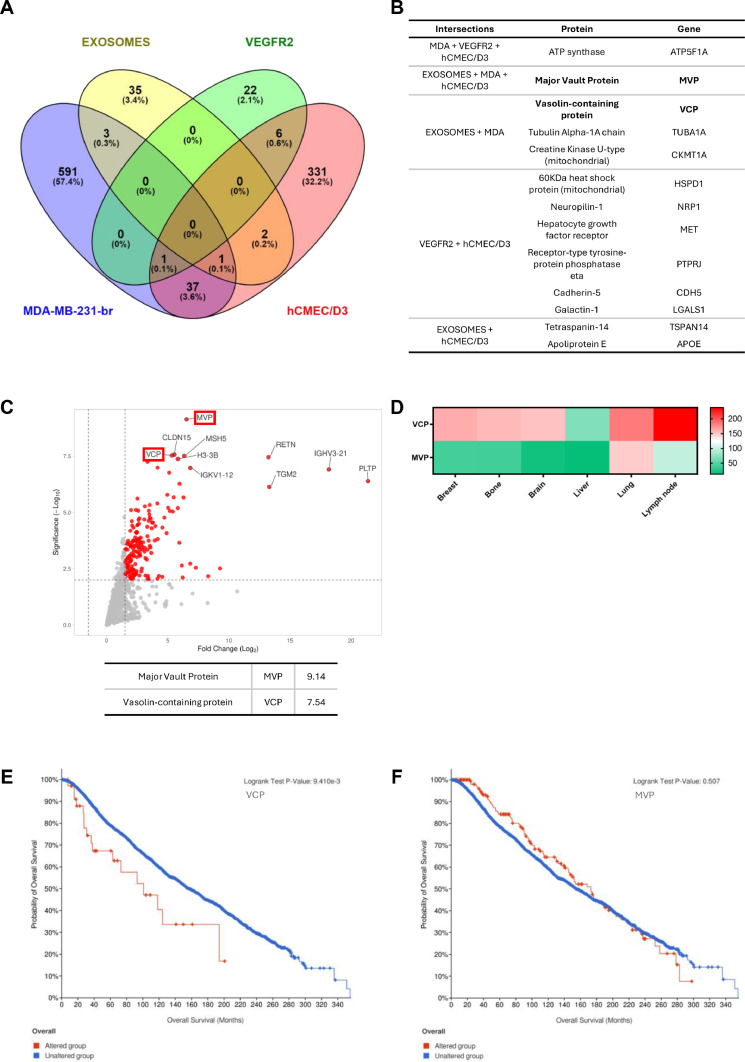
Computational analysis. **(A)** Venn diagram showing the quantity of proteins shared or not by the groups. **(B)** List of proteins identified by plotting the Venn diagram. **(C)** Volcano plot evidencing the proteins most differentially expressed in exosomes from TNBC versus healthy subjects. **(D)** RNA-seq analysis of normal tissues commonly related to metastatic sites from breast cancer (n = 122). **(E)** and **(F)** Kaplan-Meyer survival plots of 3065 patients with breast cancer and altered levels (red line) of VCP (E) (n = 37, *p=* 9.410e^-3^) or MVP (F) (n = 170, *p=* 0.507).

The raw proteomics data were processed using MaxQuant [36] and the Statistical guide workflow to Microsoft Excel [37]. After protein identification, we grouped them using a Venn diagram (Fig. 3A), and set the groups as follows: MDA_POI (Proteins of interest), consisting of proteins differentially expressed among MDA-MB-231-par (MDA-par) and MDA-MB-231-br (MDA-br); VEGFR2_Interactors, using a list of proteins interactions predicted by IntAct – Molecular Interaction Database [57]; EV_POI, proteins differentially expressed between extracellular vesicles from patients with breast cancer and healthy subjects; hCMEC, proteins identified on its cell surface.

Although we have not found proteins common to all groups, we have identified others potentially involved in the modulation of VEGFR2 phosphorylation (Fig. 3B). Thus, we verified their expression levels by graphing a Volcano plot (Fig. 3C), which allowed us to observe two proteins among the highly expressed, Major Vault protein (MVP) (fold-change = 9.14) and Vasolin-containing protein (VCP) (fold-change = 7.54). These proteins have previously been described as involved in breast tumor resistance [58] and poor survival of patients undergoing chemotherapy [59].

We then quantitatively examined the clinical relevance of VCP and MVP using an RNA-seq dataset in the Expression Atlas [60]. Higher expression of VCP than MVP was verified in all healthy tissues representing secondary sites for breast cancer metastasis (breast, brain, bone, liver, lung, and lymph node) (Fig. 3D). Therefore, we analyzed another RNA-seq dataset from patients with breast cancer [61]. Overall survival was recorded in 3065 patients over a 30-year follow-up, 37 of whom had higher VCP expression, and a lower overall survival (360 vs. 200 months, Log-rank Test p-value = 9.410 × 10^-3^) (Fig. 3E). Otherwise, 170 patients showed high levels of MVP, but no difference was found in comparison to those with no changes in this protein (360 vs. 290 months, Log-rank Test p-value = 0.507) (Fig. 3F).

### Analysis of VCP impacting VEGFR2 signaling and endocytic pathways

We hypothesized that VCP in the sEV-br might be transported to the endothelial cells. Thus, it would interact with phosphosites in VEGFR2, activating a variety of signaling pathways and promoting various cellular responses. VEGFR2 is the main regulator of endothelial functions [62] and is highly regulated by recycling and degradation machinery. For this reason, we focused on assessing the proteins involved in these processes and identifying the endocytic pathways that are prevalent in the presence of EVs.

**Fig. 4:**
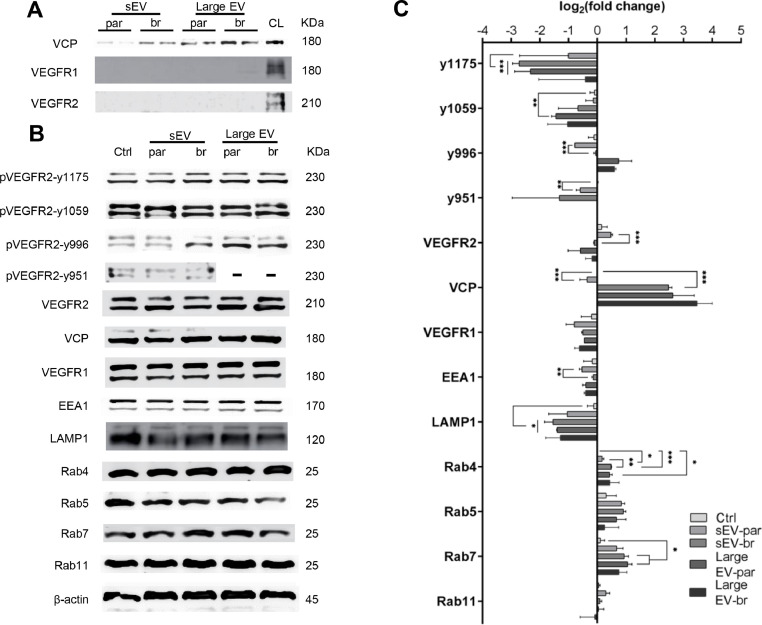
The proteins involved in VEGFR2 trafficking and its phosphosites under EVs modulation. **(A)** Western blot showing the protein expression of VCP, VEGFR1, and VEGFR2 in small and large EVs. **(B)** Representative western blot panel of different sites of VEGFR2 phosphorylation, VCP, VEGFR1, and components of endolysosomal pathway in hCMEC/D3 treated with extracellular vesicles. **(C)** Densitometry analysis of the protein bands shown in **A**, representation of Log2 Fold Change of protein level from hCMEC/D3 treated with small EV. Ctrl × sEV-par: Y951 (adj. *p=* 0.0065), Rab4 (adj. *p=* 0.0412); Ctrl × sEV-br: Rab4 (adj. *p* < 0.0001), Y1175 (adj. *p* < 0.0001), VCP (adj. *p=* 0.0006), LAMP1 (adj. *p=* 0.0196), Rab7 (adj. *p=* 0.0244); Ctrl × large EV-par: Y1175 (adj. *p=* 0.0002), Y1059 (adj. *p=* 0.0056), LAMP1 (adj. *p=* 0.0161), Rab4 (adj. *p=* adj. *p=* 0.0132), Rab7 (adj. *p=* 0.0179); Ctrl × Large EV-br: Y996 (adj. *p=* 0.0126); sEV-par × sEV-br: VCP (adj. *p=* 0.0007), Y996 (adj. *p=* 0.0007), VEGFR2 (adj. *p=* 0.0009), EEA1 (adj. *p=* 0.0095), Rab4 (adj. *p=* 0.0021); Large EV-par × Large EV-par: no significant difference. Test-t with Holm-Sidak correction, α = 0.05. Data from three independent experiments performed in triplicate (*n* = 9).

First, we measured the levels of VCP in the extracellular vesicles, finding that VCP is higher expressed in sEV-br than sEV-par. Notably, neither VEGFR1 nor VEGFR2 were carried by either of them (Fig. 4A). Subsequently, the hCMEC/D3 cells were treated with 1µg/ml of sEV-par or sEV-br, and 4 µg/ml of Large EV-par or -br, for 24h. We found almost all the phosphosites downregulated under small and large EV treatment, except the phosphosite Tyr996 (Y996) treated with large EV-par and -br. In another way, the VCP protein showed higher levels of expression among the cells treated with sEV-br, and the large EVs, while was downregulated when treated with sEV-par.

Among the regulators of small GTPases in the endocytic pathway analyzed, treatment with both small and large extracellular vesicles (EV) significantly increased Rab4 and Rab7. However, EEA1 showed consistent levels compared to the control, with the only statistically significant difference observed between cells treated with sEV-par and -br, where it was downregulated. LAMP1 was found completely downregulated compared to the control, and the total VEGFR2 protein level was only significantly different between sEV-par and sEV-br. sEV-par presented higher levels of expression, while sEV-br showed consistent levels, meaning no differences for the control (Figs. 4B and 4C).

Additionally, while NTA is not accurate in detecting particles smaller than 50nm, TEM analysis with immunogold labeling revealed the presence of vesicles above 100nm associated with VCP, suggesting that the majority of VCP is indeed associated with EVs (supplementary figure 1).

### VCP modulation in hCMEC/D3 cells

**Fig. 5:**
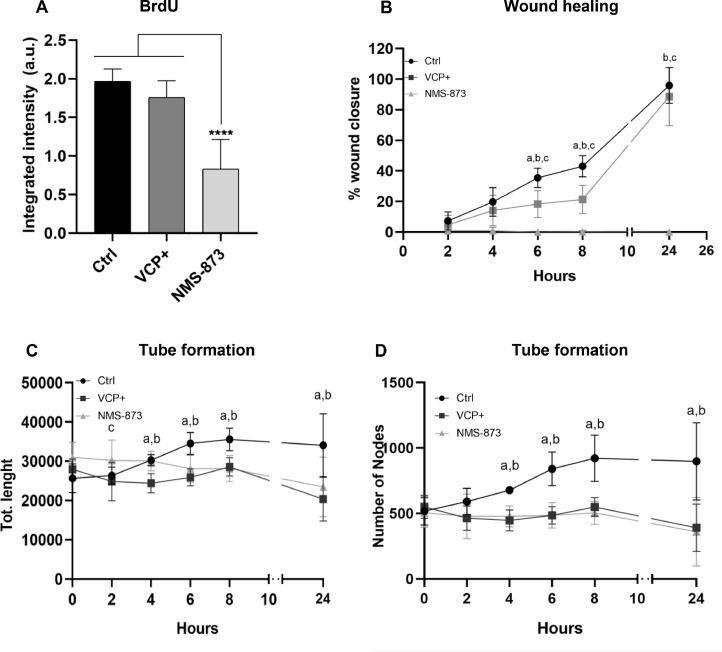
VCP modulation alters hCMEC/D3 cell function. **(A)** BrdU assay demonstrates that VCP modulation significantly reduces hCMEC/D3 cell proliferation (one-way ANOVA with Dunnett's multiple comparison test, *p* < 0.0001). **(B)** Wound healing assay showing that VCP modulation impairs hCMEC/D3 cell migration (two-way ANOVA with Dunnett's multiple comparison test, *p* < 0.0001). a = Ctrl × VCP+, b = Ctrl × NMS-873, and c = VCP+ × NMS- 873. **(C)** Tube formation assay revealed a significant decrease in total tube length in hCMEC/D3 cells upon VCP modulation (two-way ANOVA with Dunnett's multiple comparison test). a = Ctrl × VCP, b = Ctrl × NMS-873, c = VCP+ × NMS-873. **(D)** Tube formation assay demonstrated a significant decrease in the number of nodes formed in hCMEC/D3 cells upon VCP modulation (two-way ANOVA with Dunnett's multiple comparison test). a = Ctrl × VCP, b = Ctrl × NMS-873. Data from three independent experiments performed in triplicate (*n* = 9).

To evaluate the effect of VCP modulation on brain endothelial cells, different functional assays were conducted, along with a chemical inhibitor, NMS-873. Initially, the cell proliferation assay was determined by BrdU incorporation into DNA strands. Through this assay, it was observed that NMS-873 significantly reduced the proliferation of cells compared to both the control and transfect conditions. The cells overexpressing VCP observed a slight decrease in proliferation, but not statistically significant (Fig. 5A).

Next, the migration capacity of hCMEC/D3 cells was assessed using a wound-healing assay. The results indicated that when these cells were treated with the VCP inhibitor, there was a significant loss of migratory capacity toward the central gap in the experiment. Conversely, both parental and transfected cells retained their ability to migrate, displaying similar migration patterns. Notably, control cells displayed a higher propensity to completely close the gap by the 24^th^ hour, a behavior not observed in the transfected cells and less pronounced in the NMS-873-treated cells (Fig. 5B, supplementary figure 2).

Then, the capacity of hCMEC/D3 cells to form tubes mimicking an angiogenesis-like process was evaluated by seeding the cells over a Matrigel-coated well and images captured at different time points throughout 24h. The findings revealed that both transfected and NMS-873 treated cells had a significant decrease in the total tube length (Fig. 5C) and number of nodes formed (Fig. 5D), highlighting the VCP-potential roles in modulating angiogenesis-related processes (Supplementary material figure 3).

**Fig. 6:**
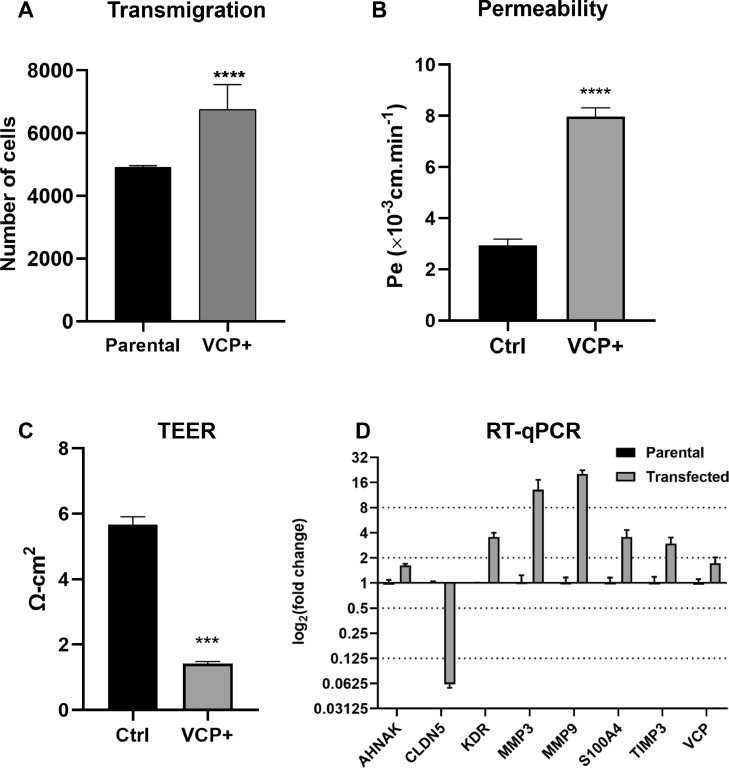
**VCP modulation impairs cell barrier properties and gene expression. (A)** Transmigration assay demonstrating that VCP overexpression significantly increases breast cancer cell transmigration across the hCMEC/D3 cell monolayer (unpaired t-test with Welch's correction, *p* < 0.0001). **(B)** FITC permeability assay shows that VCP overexpression significantly increases endothelial cell permeability (unpaired t-test with Welch's correction, *p* < 0.0001). **(C)** TEER measurement demonstrates a significant decrease in TEER values of hCMEC/D3 cell monolayer upon VCP modulation (unpaired t-test with Welch's correction, *p* < 0.0001). **(D)** RT-qPCR analysis of gene expression in hCMEC/D3 cells overexpressing VCP. The horizontal line represents a p-value threshold of 0.01, indicating statistically significant differences between the group mean and its respective control. Data from three independent experiments performed in triplicate (*n* = 9).

The ability of a brain endothelial cell monolayer to maintain its barrier function was evaluated through a transmigration and permeability assay, and TEER measurement. As shown in Fig. 6A, cells overexpressing VCP exhibited significantly higher permeability to breast cancer cells passing through the pores compared to the control cells. Concomitantly, there was a significant increase in the FITC amount permeating through the membrane pores compared to the control (Fig. 6B), as well as decreased resistance values in the TEER measurement (Fig. 6C).

Given that VCP can influence gene regulation, different targets were assessed for their expression levels (Fig. 6D). Claudin-5 was the most significantly downregulated gene, suggesting a potential disruption of the BBB function. Genes such as MMP9 and MMP3 showed significant upregulation, indicating a higher chance of cell detachment from the basement membrane. Other genes related to BBB maintenance, like AHNAK, S100A4, and KDR (VEGFR2), also exhibited notable upregulation, hinting at alterations in barrier properties in the transfected cells.

Taken together, these results suggest that VCP overexpression may compromise the integrity of the endothelial barrier, modulating gene expression and cellular function, leading to increased permeability and transmigration of breast cancer cells.

### Proposed model of VCP influencing brain endothelial barrier disruption

**Fig. 7:**
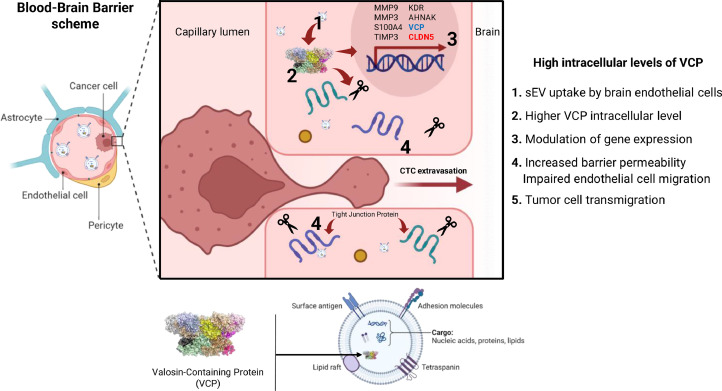
Proposed model of VCP-mediated blood-brain barrier disruption and breast cancer brain metastasis. Breast cancer cells, especially those with brain tropism, release small EVs enriched with VCP. These sEVs are internalized by brain microvascular endothelial cells (hCMEC/D3), leading to VCP accumulation and downstream effects that disrupt BBB integrity. These effects include increased permeability, impaired angiogenesis, and altered gene expression favoring cell motility and extracellular matrix degradation while compromising barrier maintenance. The weakened BBB allows CTCs to transmigrate into the brain parenchyma, ultimately resulting in the establishment of brain metastases.

Taken together, our results suggest a mechanistic model (Fig. 7) where tumor-derived sEV enriched with VCP contributes to BBB disruption and breast cancer brain metastasis. Upon internalization by hCMEC/D3 cells, VCP accumulation triggers downstream effects compromising BBB integrity, including disruption of tight junction complexes, increasing permeability; impaired angiogenesis, hindering BBB repair; and modulation of gene expression, favoring cell motility and ECM degradation while downregulating barrier maintenance genes. This VCP-mediated BBB disruption facilitates CTC transmigration into the brain parenchyma, leading to metastasis.

## Discussion

This study sheds light on the critical role of tumor-derived sEV in reprogramming the brain endothelial cell environment, ultimately promoting brain metastasis establishment. Our findings identify VCP as a key player in disrupting the endothelial monolayer phenotype, with significantly higher levels observed in sEV derived from brain-tropic breast cancer cells compared to their parental counterparts. This observation aligns with the emerging recognition in the tumor microenvironment, influencing the recipient cells’ behavior and contributing to metastatic progression [63].

Investigating the protein expression levels via western blot, we observed a decrease in total VEGFR2 in hCMEC/D3 cells treated with sEV-br compared to sEV-par, while VEGFR1 levels remained unchanged. These findings are consistent with previous studies demonstrating that VEGFR2 is modulated by several factors affecting its quantity, availability, and activity [30]. It is noteworthy that despite lower total VEGFR2 protein levels, we observed an overall increase in its phosphorylation in hCMEC/d3 cells treated with sEV-br, suggesting a potential shift towards a more active signaling state. This observation aligns with the concept of VEGFR1 acting as a homeostatic regulator of VEGFR2 [64], potentially counterbalancing the effects of sEV-br on VEGFR2 expression and activity.

However, the direct binding of factors like VEGFR to the VEGFR2 extracellular domain is not mandatory for its activation. Non-canonical mechanisms, including ligand-independent activation, are highly relevant [65]. Thus, we explored public proteomic and transcriptomics datasets, identifying VCP as a possible interactor of VEGFR2. This finding aligns with previous studies demonstrating the involvement of VCP in various cellular processes, including receptor trafficking and endocytosis [66]. Importantly, we validated the presence of VCP in tumor-derived sEV through western blot analysis, suggesting a potential mechanism for its delivery to endothelial cells.

Recognizing the multifaceted functionality of VCP beyond VEGFR2 regulation, we investigated its broader impact on hCMEC/D3 cell behavior. We observed a trend towards decreased proliferation in VCP-overexpressing hCMEC/D3 cells, although not statistically significant, in contrast to studies reporting increased proliferation in other cell types [67,68]. This discrepancy might be attributed to cell type-specific differences in VCP function or variations in experimental conditions. However, VCP inhibition by NMS-873 significantly reduced hCMEC/D3 cell proliferation, highlighting VCP's essential role in maintaining normal endothelial cell function.

Further investigation into VCP's influence on endothelial cell function revealed significant impairment of wound healing capacity in VCP-overexpressing hCMEC/D3 cells, with complete abolishment of cell migration upon VCP inhibition. This observation contrasts with the known role of endothelial cells in promoting wound healing [69,70]. This contrasting effect could be attributed to VCP's potential influence on Endothelial-Mesenchymal Transition (EndMT), a process implicated in wound healing and characterized by the downregulation of endothelial markers and acquisition of a mesenchymal phenotype [71]. VCP's dysregulation might disrupt the delicate balance of EndMT, leading to impaired wound healing and potentially facilitating extravasation of CTCs, as observed in melanoma [72].

Furthermore, we observed increased permeability in VCP-overexpressing hCMEC/D3 monolayers, evidenced by both the FITC permeability assay and reduced TEER measurements. This finding corroborates previous studies demonstrating the impact of various signaling pathways, such as Wnt signaling, on BBB permeability [73]. It is intriguing to note that Wnt signaling pathways intersect with VCP activity [74,75], suggesting a potential link between VCP dysregulation and compromised endothelial barrier function in various pathological conditions.

VCP's role in promoting metastasis, particularly its influence on barrier disruption, has been reported in other cancer types. In non-small cell lung carcinoma, VCP interacts with MEST, leading to NF-kB pathway activation, promoting tumor invasion and metastasis [68]. Additionally, VCP has been implicated in promoting EMT through the TGF-β1/Smad2/3 signaling pathway, while its inhibition attenuates osteosarcoma cell invasion and metastasis [76]. These studies, along with our findings in the transmigration assay, where VCP overexpression significantly facilitated breast cancer cell passage across the endothelial monolayer, collectively highlight VCP's critical role in promoting tumor cell extravasation across various endothelial barriers.

Delving deeper into the molecular mechanisms underlying VCP's influence on brain endothelial cells, we analyzed the expression of various genes related to BBB function. The significant upregulation of MMP3 and MMP9 genes in VCP-overexpressing hCMEC/D3 cells suggests a potential role for VCP in modulating extracellular matrix dynamics and tissue remodeling processes, crucial for angiogenesis and cancer metastasis. This finding aligns with studies demonstrating MMP-3 and MMP-9 involvement in degrading the neurovascular basal lamina and tight junction proteins, contributing to BBB disruption [74,77].

Furthermore, the upregulation of S100A4 and AHNAK, known to regulate cell motility and invasion [[78], [79], [80]], in VCP-overexpressing hCMEC/D3 cells emphasizes VCP's role in promoting metastasis by disrupting endothelial cell function. Previous studies have linked S100A4 to increased BBB permeability through occludin downregulation [81], further supporting its contribution to VCP-mediated BBB disruption.

Finally, the observed alteration in the expression of KDR (VEGFR2) and Claudin5 in VCP-overexpressing hCMEC/D3 cells underscores the influence of VCP on endothelial cell proliferation and barrier maintenance. The upregulation of KDR is consistent with the previously discussed role of VCP in regulating angiogenesis and vascular permeability and highlights its involvement in pathological conditions characterized by aberrant angiogenesis, such as in cancer and vascular diseases [[82], [83], [84]]. Conversely, the dysregulation of Claudin5, a critical component of endothelial cell junctions, further supports VCP's involvement in disrupting endothelial barrier integrity [85], potentially contributing to increased vascular permeability and disease progression.

## Conclusions

This study provides compelling evidence for a novel role of VCP in promoting breast cancer brain metastasis through its impact on BBB integrity and function. We demonstrate that brain-tropic breast cancer cells release VCP-enriched sEV. Treatment of brain microvascular endothelial cells with these sEVs or overexpression of VCP in endothelial cells resulted in the accumulation of VCP in brain endothelial cells. It triggered downstream effects that compromised the integrity of the BBB. These effects included increased permeability, impaired angiogenesis, and a shift in gene expression patterns favoring cell motility and ECM degradation while compromising barrier maintenance. Our findings suggest that VCP represents a potential therapeutic target in breast cancer. Further research is needed to elucidate the molecular mechanism underlying VCP-mediated BBB disruption fully and to evaluate the efficacy of VCP-targeted therapies in preclinical models of breast cancer brain metastasis.

## Ethics declarations

Not applicable.

## Consent for publication

Not applicable.

## Funding

This research was funded by FAPESP, grant number 2020/15696-4. RGT and NJV received CAPES scholarship, partially financed in part by the Coordenação de Aperfeiçoamento de Pessoal de Nível Superior – Brasil (CAPES) – Finance Code 001, RGT was funded by CAPES-DAAD (57588368), ASY received FAPESP (# 2020/15751-5).

## CRediT authorship contribution statement

**Ramon Handerson Gomes Teles:** Writing – review & editing, Writing – original draft, Visualization, Validation, Methodology, Investigation, Formal analysis, Data curation, Conceptualization. **Nicolas Jones Villarinho:** Writing – original draft, Methodology, Investigation. **Ana Sayuri Yamagata:** Writing – original draft, Methodology, Investigation. **Camila Tamy Hiroki:** Writing – original draft, Methodology. **Murilo Camargo de Oliveira:** Methodology. **Gisela Ramos Terçarioli:** Methodology. **Ruy Gastaldoni Jaeger:** Writing – original draft, Funding acquisition. **Patrick Meybohm:** Writing – review & editing, Writing – original draft, Resources, Funding acquisition. **Malgorzata Burek:** Writing – review & editing, Supervision, Resources, Project administration, Conceptualization. **Vanessa Morais Freitas:** Writing – review & editing, Supervision, Resources, Project administration, Funding acquisition, Conceptualization.

## Declaration of competing interest

The authors declare that they have no known competing financial interests or personal relationships that could have appeared to influence the work reported in this paper.

## Data Availability

The datasets used and/or analyzed during the current study are available from the corresponding author on reasonable request.

## Appendix Supplementary materials

Image, application 1
