# Supplementary material for: Valosin-containing protein (VCP), a component of tumor-derived extracellular vesicles, impairs the barrier integrity of brain microvascular endothelial cells
Source: BBA Adv. 2024 Dec 12;7:100130. doi: 10.1016/j.bbadva.2024.100130 (PMC11722580; doi:10.1016/j.bbadva.2024.100130)
Supplement: Supplementary file 1 [file mmc1.docx]

Supplementary Methods

Immunogold labeling of extracellular vesicles by transmission electron microscopy

The protocol used was modified from (Capomaccio et al., 2019). After isolation, SEVs were diluted in a proportion 1:20 in filtered PBS. Then, the SEVs solution was adsorbed onto a formvar- and carbon-coated nickel grid (Koch Electron Microscopy LTDA. FCF200-400 MESH-NI-50) for 1h at room temperature. Excess liquid was removed with filter paper, and the sample was blocked in 10% donkey serum in PBS for 10min. The grids were washed twice for 2min with 1% bovine serum albumin (BSA) in filtered PBS (0.22 µm pores). The grids were incubated in 20 µL of anti-VCP (Cell Signaling, Cat. 2648S) or anti-TSG101 (Cell Signaling, Cat. HPA006161) diluted 1:50 in 1% PBS BSA in a humid chamber at room temperature overnight. The grids were then washed 8 times for 2min in PBS, and incubated in 20 µL of 18 nm Colloidal Gold AffiniPure™ Goat Anti-Rabbit IgG (H+L) (EM Grade) (Jackson ImmunoResearch 111-215-144) diluted 1:10 in 1% PBS BSA for 2h at room temperature in a humid chamber. The grids were washed 8 times for 2min, fixed in 2.5% glutaraldehyde for 10min, washed 3 times for 2min, and dried for 1h at room temperature. Contrast was applied using 2% uranyl acetate for 5min. Micrographs were captured using a Jeol 1010 transmission electron microscope. The vesicles were observed at a magnification of 5,000 and 10,000x.

Supplementary material figure 1


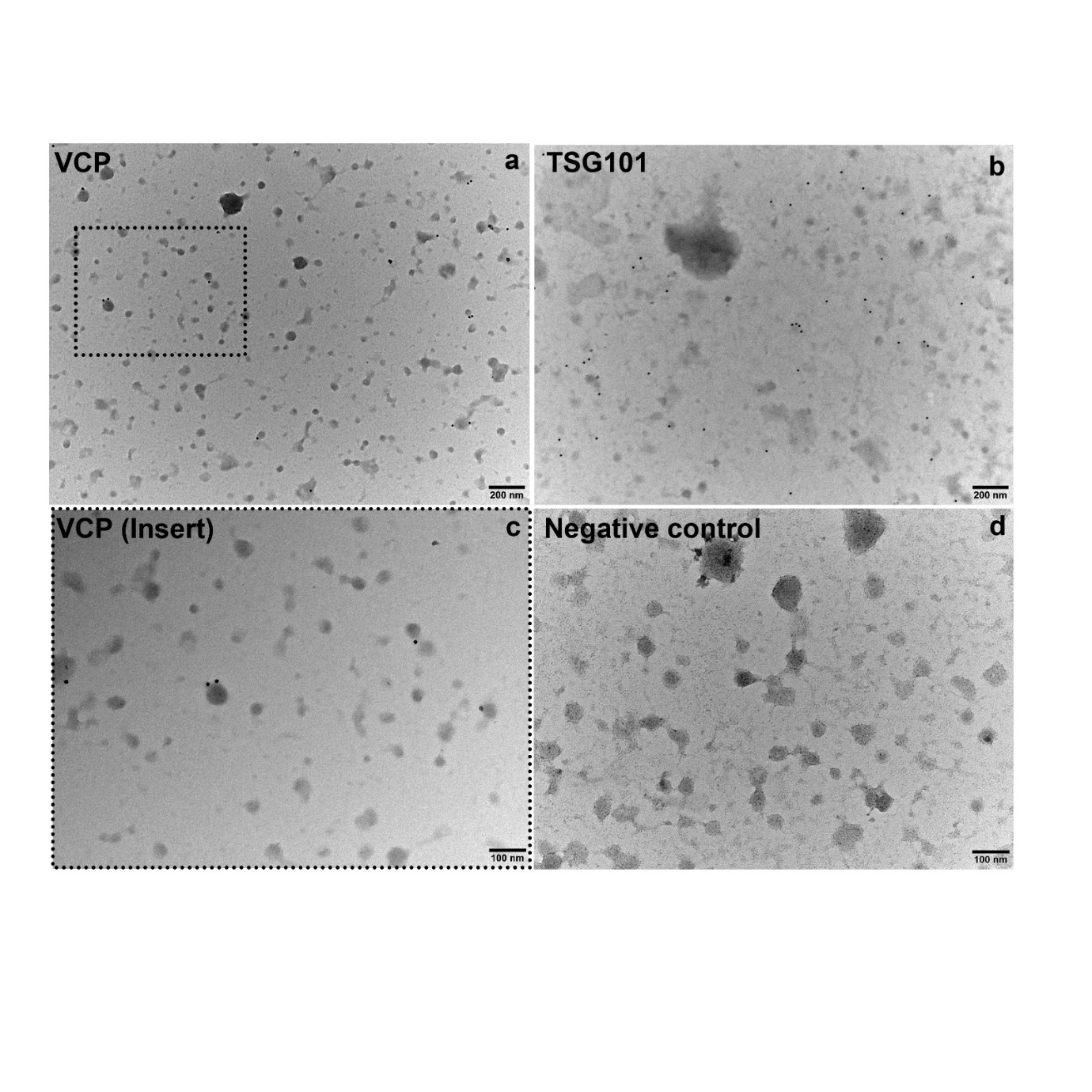


VCP is present in sEVs derived from MDA-MB-231-BR cells. VCP localization in extracellular vesicles is demonstrated by immunogold labeling (a and c, insert). TSG101 (positive control) and anti-rabbit colloidal gold (negative control) are shown in panels b and d, respectively. Notably, VCP is detected exclusively in extracellular vesicles and not in protein aggregates.

Supplementary material figure 2


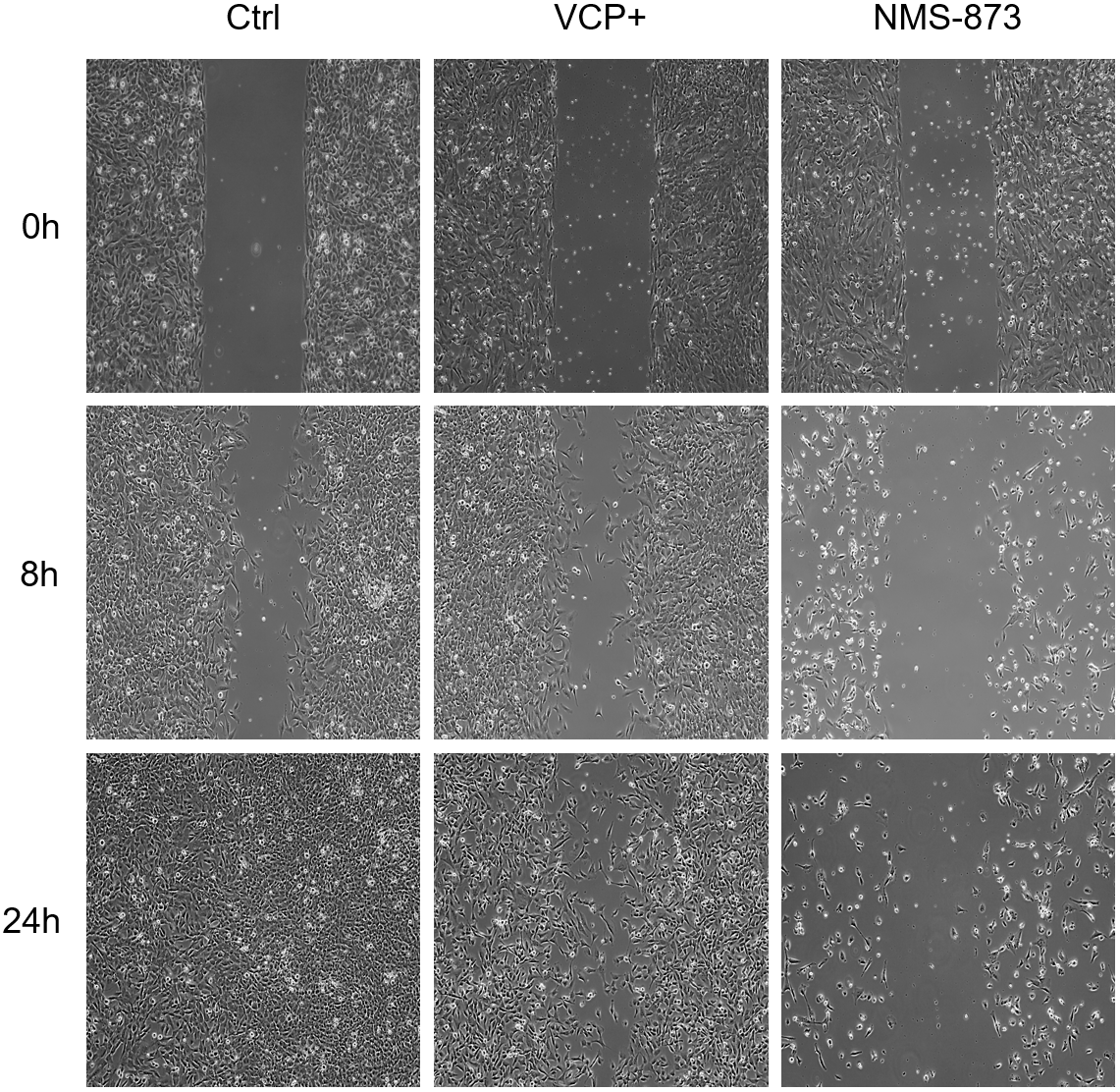


**VCP modulation impairs hCMEC/D3 cell migration in a wound healing assay.** Representative images captured at 0, 2, 4, 6, and 24 hours after introducing a scratch wound in confluent hCMEC/D3 monolayers analyzed in Figure 5B. (A) Control (Ctrl) cells exhibit robust migration into the wound area over time, leading to significant wound closure. (B) Cells overexpressing VCP (VCP+) display delayed and less efficient wound closure compared to control cells. (C) Treatment with the VCP inhibitor NMS-873 severely impairs cell migration, resulting in minimal wound closure even after 24 hours. These observations emphasize the crucial role of VCP in regulating hCMEC/D3 cell motility and wound healing capacity.

Supplementary material figure 3

**VCP modulation impairs angiogenesis-like tube formation in hCMEC/D3 cells.** Representative images of hCMEC/D3 cells cultured on Matrigel for 2, 4, 6, 8, and 24 hours, demonstrating the formation of capillary-like structures and analyzed in Figure 5 C and D. (A) Control (Ctrl) cells display robust tube formation over time. (B) Cells overexpressing VCP (VCP+) exhibit reduced tube formation and network complexity. (C) Treatment with the VCP inhibitor NMS-873 significantly impairs tube formation, resulting in fragmented and disorganized structures. These observations highlight the critical role of VCP in regulating angiogenesis-like processes in brain endothelial cells.

**
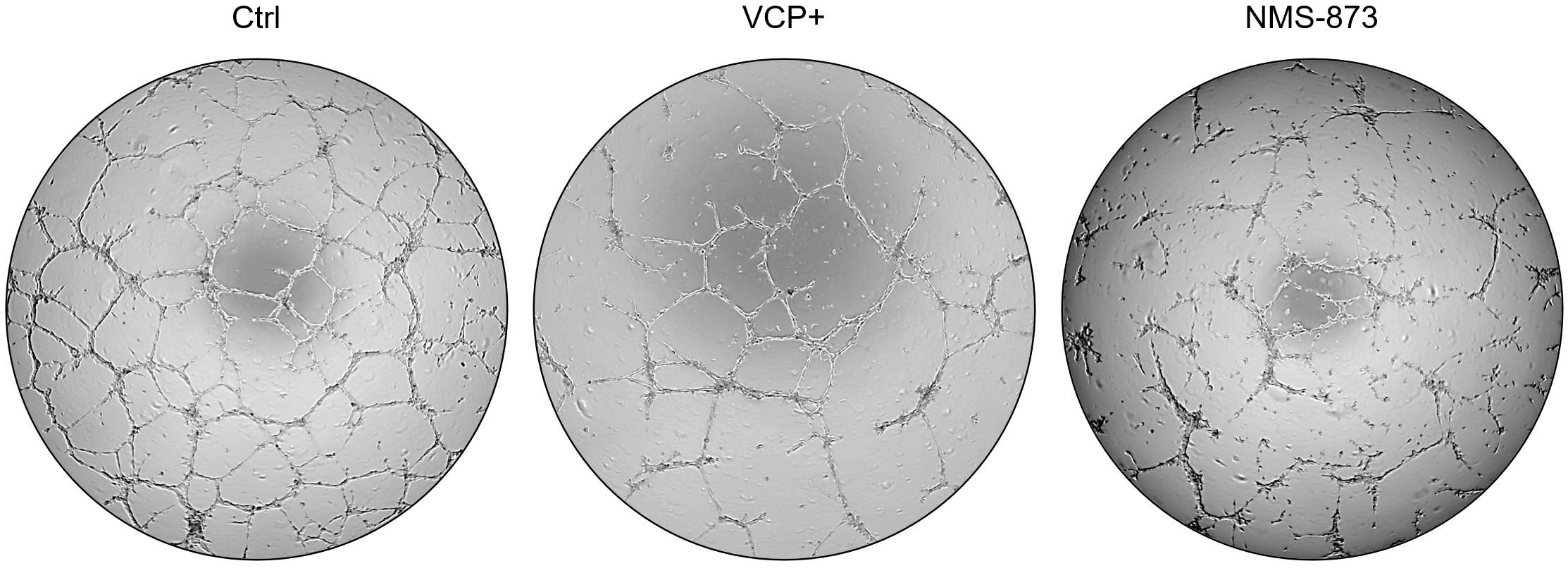
**
